# Psychotherapy and change in mental health-related work disability - A prospective population level register-based study in Finland

**DOI:** 10.1093/eurpub/ckac129.682

**Published:** 2022-10-25

**Authors:** J Kausto, K Gluschkoff, S Selinheimo, J Turunen, A Väänänen

**Affiliations:** Finnish Institute of Occupational Health, Helsinki, Finland

## Abstract

**Introduction:**

Mental disorders are a major cause of work disability among working age population. Psychotherapy has been shown to be an effective treatment for mental disorders, but the evidence mainly comes from small scale randomised trials with a relatively short follow-up.

**Objectives:**

We used population-based register data to examine the association between statutory rehabilitative psychotherapy and change in depression or anxiety related work disability using a quasi-experimental interrupted time series analysis.

**Methods:**

All those who started rehabilitative psychotherapy in 2011-2014 comprised the study group. The study group included 10436 participants who were followed from three years prior to four years after the onset of rehabilitative psychotherapy, resulting in 83488 observations. Annual total number of mental health related work disability months was calculated based on total number of annual compensated sickness absence and disability pension days.

**Results:**

The onset of rehabilitative psychotherapy marked a decline in depression or anxiety related work disability as compared to the counterfactual trend. Specifically, a 20% decrease in the level (incidence rate ratio, IRR 0.80; 95% CI 0.76-0.85) as well as a 48% decrease in the slope (IRR 0.52; 95% CI 0.50-0.54) of work disability was detected. The decline was steepest in the oldest age-group

**Conclusions:**

Providing statutory psychotherapy may decrease work disability at the population level. Further evidence for causal inference and the potential heterogeneity of the association is required.

